# Direct Nerve Sutures in (Extended) Upper Obstetric Brachial Plexus Repair

**DOI:** 10.1055/s-0037-1608624

**Published:** 2017-11-09

**Authors:** J. Bahm, A. Gkotsi, S. Bouslama, W. El-kazzi, F. Schuind

**Affiliations:** 1 Euregio Reconstructive Microsurgery Unit, Franziskushospital Aachen, Aachen, Germany; 2 Department of Orthopaedics and Traumatology, Université Libre de Bruxelles, Erasme University Hospital, Brussels, Belgium

**Keywords:** brachial plexus injury, obstetric brachial plexus palsy, microsurgery, nerve repair, direct suture, tension

## Abstract

**Background** In rare, selected cases of severe (extended) upper obstetric brachial plexus palsy (OBPP), after supraclavicular exposure and distal mobilization of the traumatized trunks and careful neuroma excision, we decided to perform direct nerve coaptation with tolerable tension and immobilized the affected arm positioned in adduction and 90-degree elbow flexion for three weeks.

**Objectives** We present our surgical technique and preliminary results in a prospective open patient series, including 22 patients (14 right and 8 left side affected) between 2009 and 2016, operated at a mean age of 8.4 months.

**Methods** Analysis of functional results after a minimum of 18 months was conducted using the British Medical Research Council (BMRC) scale.

**Results** All children reached 60–90° of elbow flexion and 75° of shoulder abduction at already six months after surgery. For those patients having already passed one year post surgery, the mean active shoulder abduction reached 92°, and for those who past the 18 months 124°. We discuss the actual knowledge about nerve coaptation under “reasonable” tension including its advantages and drawbacks.

**Conclusion** This technique may be indicated in preoperatively selected cases of (extended) upper OBPP and may give good functional results.

## Introduction

Severe upper and total obstetric brachial plexus palsies (OBPPs) need early surgical exploration and microsurgical reconstruction. 1 The brachial plexus lesion is routinely exposed through a transverse supraclavicular approach and consists most often in root ruptures and/or avulsion(s) associated with neuroma formation in the supra- and retroclavicular space. Although root avulsions may not be addressed surgically and repaired directly in young children, if two or three root stumps remain, an intraplexic reconstruction after resection of the neuroma with autologous bridging grafts is most often feasible. Extraplexic nerve transfers might be added.

After the resection of the neuroma which usually extends over 1 to 2.5 cm, the proximal and distal stumps should be controlled histologically and then autologous grafts are interposed and anastomosed both proximally and distally, using microsurgical sutures (8 to 10/0) and/or fibrin glue.

The use of grafts needs autologous nerve harvest, most often at the leg (sural nerve), associated with a donor defect (scar, loss of sensibility).

In total lesions, the lower trunk is often conserved in its length as the roots C8 and T1 are frequently avulsed, and then it might be sutured directly to a remaining root, either C7 or C6. It is also sometimes possible to reconnect the middle trunk directly, after excision of a short neuroma.

These observations help us identify specific cases of upper lesions, where extensive distal, retroclavicular dissection of the affected trunks and their proximal mobilization allowed direct suture of the upper and/or middle trunk to the original root stumps, thereby realizing a microsurgical reconstruction with single coaptation sites and without donor nerve morbidity.

We present our surgical technique and preliminary results from a prospective patient series.

## Surgical Technique

**Fig. 1:**
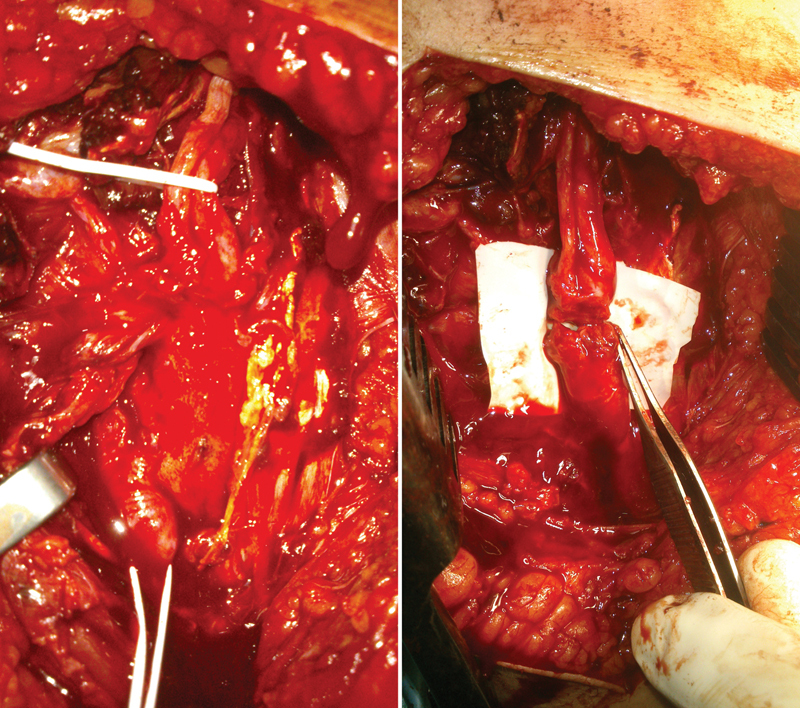
Trimmed nerve stumps of a radial nerve entrapment injury before and after direct coaptation.

In some clinical cases of peripheral nerve injury, especially of radial nerve repair at the humeral shaft level, we encounter proper nerve stumps with a short gap (around 1 cm, Fig. 1 ) needing either short grafts or a technique of distal stump advancement to perform a direct nerve suture. As direct sutures with 8/0 epineural anastomosis provided excellent clinical recovery despite some tension, we tried to apply this technique to the reconstruction of OBPP.

There is, of course, no possibility of preoperative selection. Only when the lesion has been exposed and shows two or three good upper root stumps, and a rather short neuroma (macroscopically 1.5 cm maximum), we investigate the possibility to bring the distal nerve parts more proximally using different technical tricks.

**Fig. 2:**
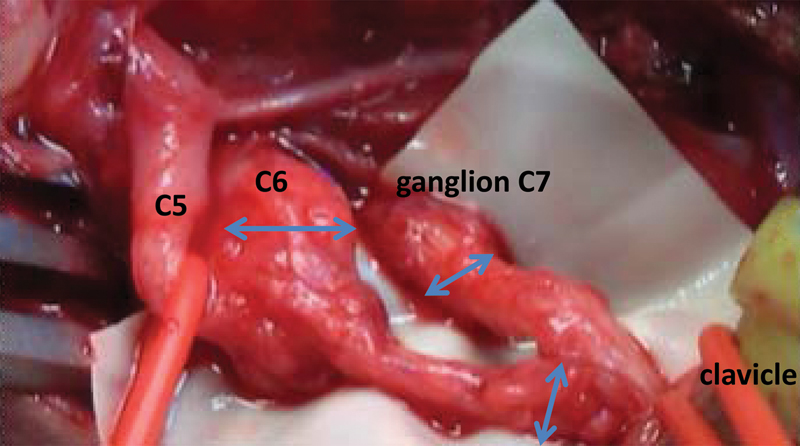
Intraoperative situation before neuroma excision and possible coaptation of upper and middle trunk.

First, the distal trunks and cords are dissected as far distally as possible in the retroclavicular area and even in the infraclavicular area (through the supraclavicular incision only); therefore, the clavicle is looped with an aspiration silicone tube and all adhesions around the traumatized nerves are freed in the retro- and infraclavicular spaces ( Fig. 2 ). The upper extremity is brought into elbow flexion and shoulder adduction, and the upward movement of the distal nerve stumps close to the proximal parts is analyzed. We always take histologic samples from the proximal and distal stumps to make sure no major (perineural) scar or neuroma (the presence of minifascicles) is left—which would preclude proper reinnervation.

Then, the suture is tried with isolated 8/0 epiperineural sutures and a microsurgical grasp holding the distal nerve part until the first one or two sutures are accomplished. If these do not rupture, the anastomosis is performed circumferentially (six or eight sutures to decrease the local tensile strength) and is finally wrapped with fibrin glue (Tisseel 2 mL Baxter). The other coaptations are performed in the same way and after bringing back the adipofascial flap, the wound is closed and the arm is maintained flexed and adducted with the head and neck in a plaster immobilization for 2 weeks.

After this period, the head is freed and the arm is maintained in elbow flexion of 90 degrees for 1 more week. Then the restriction of passive motion is abandoned and the physiotherapy started again.

## Materials and Methods

Between 2009 and 2016, we identified 22 out of 140 operated OBPPs reconstructed by direct nerve coaptation, initially suffering from an upper lesion (14 right, 8 left) operated at a mean age of 8.4 months (1 exception being operated at 3 months of age, due to a subtotal lesion with Horner's sign).

Nine underwent surgery in 2016, four in 2015, two in 2014, four in 2013, and the rest before 2012.

There were:

- Fourteen cases of combined ruptures of the upper and middle trunk segment, including lesions in the distal radicular area. Among them we found three cases associated with a C8 root rupture.
- Seven cases of isolated upper trunk lesions, one of them associated with a C8 root rupture.
- One case of partial upper trunk lesion.

The maximum length of neuroma excised was 15 mm, allowing for direct suture after stump mobilization.

The following reconstructions were performed:

- Seven anatomic reconstructions by direct sutures of the upper and middle trunk.
- Thirteen reconstructions of the upper trunk alone (six of which were anatomic and seven were partially anatomic, involving a direct suture between the fifth and sixth roots with the upper trunk).
- Two anatomic reconstructions by direct sutures of the middle trunk.

Among the partially anatomic reconstructions of the upper trunk, we performed associated complete or fascicular intraplexic nerve transfers from the fifth or sixth root, participating in the direct suture with the middle trunk in three cases and with the distal C8 root in one case. We also found two cases of anatomic reconstruction of the C8 root by direct suture and one more case of direct suture between C7 root and C8 root. The two cases of anatomic reconstruction of the middle trunk alone were associated with a graft reconstruction of the upper trunk.

Extraplexual neurotization of the suprascapular nerve by the distal branch of the accessory nerve was performed in 11 of our cases, given that the beginning of the suprascapular nerve was lost with the neuroma excision.

Recently, we were able to perform the same technique in a 15-year-old adult with fresh traumatic brachial plexus injury due to a motorcycle accident (including complete rupture of the roots C5, C6, and C7).

All patients were immobilized within 2 weeks and then underwent regular physiotherapy and postoperative functional evaluation at 6-month intervals, testing active and passive range of motion.

## Results

Functional recovery started 4 months after the surgery. We were impressed by the high-quality improvement, especially at the shoulder and elbow levels ( Videos 1 and 2 of two different patients were taken 9 months after the surgery). The active shoulder abduction and flexion range obtained seemed to be better compared with all graft results we have seen in 15 years, even when slight coactivation pattern could be identified.

**Video 1** Functional result in an obstetric brachial plexus palsy 9 months after direct suture of upper and middle trunk. Online content including video sequences viewable at: http://www.thieme-connect.com/products/ejournals/html/doi/10.1055/s-0037-1608624 .

**Video 2** Second patient. Online content including video sequences viewable at: http://www.thieme-connect.com/products/ejournals/html/doi/10.1055/s-0037-1608624 .

All patients were assessed regularly measuring shoulder abduction/external rotation and elbow flexion.

Our preliminary results are the following:

- All children attained 60 to 90 degrees of elbow flexion 6 months after the surgery and their mean shoulder abduction was of 75 degrees at the same time elapse.
- The mean active shoulder abduction reached 92 degrees 1 year after surgery and 124 degrees after 18 months. For the same group of patients, the mean external rotation ranged between 60 and 90 degrees.

In two cases with pure upper trunk reconstruction without addressing specifically shoulder lateral rotation, a medial rotation motion pattern with limited passive lateral rotation of the glenohumeral joint with the arm in adduction was observed; so, we recommend in these cases to associate systematically with an extraplexic neurotization of the suprascapular nerve by the distal branch of the accessorius XIth cranial nerve to create immediately a counteracting lateral rotation force.

## Discussion

Direct sutures in obstetric brachial plexus lesions have been described for over 100 years 2 and are part of the reconstruction strategy in other OBPP teams. 3 We hereby present our series of (extended) upper lesions with short neuroma excision and intended direct repair as described earlier.

The direct suture technique has clear advantages: no grafts are needed; fast (and probably stronger) recovery through dense nerve fascicles; and “anatomical” reconstruction.

The drawbacks are that one should accept some tension at the coaptation site (elbow flexion 90 degrees) 4 and some neuroma/minifascicles at the transsection and coaptation level might be responsible for some neuromatous coactivations.

To our knowledge, there are only eight studies 5 6 7 8 9 10 11 12 that discuss suture under tension which were performed on rats, cats, dogs, and monkeys, using sciatic or upper limb nerves, and studying the outcome by histology and nerve conduction. There are so far no conclusive data about what is better and how much tension is tolerated: some surgeons use the test of 10 or 9/0 filament rupture to decide about a direct repair.

The limitations of this technique are the distance between the nerve ends and the distal dissection achieved, offering the necessary length for a loose suture. However, attention should be paid to the slight difference between tension free and loose suture. A very close coaptation might lead to axonal malalignment because of the great interference of intraneural connective tissue, while a loose coaptation would allow the endoneural tissue to protrude from the fascicles.

In addition, it should be kept in mind that healthy nerve tissue, proximally and distally to the suture site, is well extensible, being able to compensate for short defects, under the condition of being fully freed. Millesi has already expressed his confidence in this inner nerve extensibility, presenting first the possibility of a direct neurorrhaphy taking place in spite of a relatively large gap by flexing the adjacent joints, in a final effort to decrease tension.

As always, the inability to organize a statistical analysis to support a surgical technique is a vulnerable point for its spread. The great variability and individuality of the lesions encountered, as well as the rarity of cases, render a potential categorization or classification of the lesions very difficult, if not impossible. What is more, the surgical reconstruction performed is individualized and adapted to the lesions encountered. Therefore, a statistical analysis of our results is not feasible. Finally, surgical capacity is another important limitation affecting the outcome of this technique.

## Conclusion

Severe OBPP lesions are defined only after a thorough supraclavicular exploration. The assessment of the proximal root quality (by electrical stimulation and neuropathology) is possible and mandatory. From dense fiber reconstruction by multiple grafts or direct coaptation, we would expect enhanced muscle strength.

Concerning the technique of direct sutures in microsurgical OBPP repair, there remains questions about the tolerable tension (see the mechanical–histologic studies by Trumble 4 ) and the importance of minimal residual neuroma at the proximal or distal nerve stumps. Nevertheless, the good clinical results in these selected cases encourage us to further develop and promote this technique in peripheral nerve reconstruction.
